# Randomized controlled trial for anesthesia during gastroscopy: interactions between remimazolam and propofol in combination with sufentanil

**DOI:** 10.1007/s11096-023-01568-y

**Published:** 2023-04-13

**Authors:** Song Lyu, Qingchung Deng, Weixin Lin, Xiaofang Wu

**Affiliations:** 1 Department of Anesthesiology, The Second Affiliated Hospital of Hainan Medical University, Haikou, China; 2 Department of Gynecology, The Second Affiliated Hospital of Hainan Medical University, Haikou, China

**Keywords:** Drug combinations, Drug synergism, Gastroscopy, Propofol, Remimazolam, Sufentanil

## Abstract

**Background:**

Remimazolam is a new short-duration anesthetic currently used for gastroscopy and can be mixed with propofol and potent opioids.

**Aim:**

The study aimed to investigate the synergistic interaction between remimazolam and propofol after sufentanil administration and to determine the appropriate dose ratios between remimazolam and propofol.

**Method:**

This study used a randomized controlled design. Patients scheduled for gastrointestinal endoscopy were included and randomized into five groups. The randomized block design was applied at a randomization ratio of 1:1. Patients in each group received sufentanil (0.1 μg/kg) and the calculated doses of remimazolam and propofol. Using the up and down method, the median effective dose (ED_50_) and the 95% confidence interval (CI) were determined based on whether the eyelash reflex disappeared in each treatment group. Isobolographic analysis was used to analyze the presence of drug interactions. The interaction coefficient and the dose ratio between remimazolam and propofol were calculated by algebraic analysis. Statistical analysis was performed using interval estimates and 95% CI for statistical attributes.

**Results:**

Cross-sectional analysis of the isobologram showed a clinically significant synergistic effect between remimazolam and propofol. When 0.016, 0.032, and 0.047 mg/kg of remimazolam were combined with 0.477, 0.221, and 0.131 mg/kg of propofol, the interaction coefficients were 1.04, 1.21, and 1.06, respectively. The dose ratio of remimazolam to propofol was approximately 1:7.

**Conclusion:**

Remimazolam and propofol have synergistic clinical effects. A strong synergistic effect was observed when the remimazolam and propofol dose ratio was 1:7 (mg/kg).

**Clinical trial:**

The study protocol was registered at the Chinese Clinical Trial Registry (ChiCTR2100052425).

## Impact statements

- Anesthetic drugs often interact synergistically to achieve a greater sedative effect at lower doses and reduced concentration-dependent side effects. An optimal dose ratio should be selected to achieve the desired synergistic impacts.
- The dose ratio of remimazolam to propofol administered by injection should be approximately 1:7 when combined.

## Introduction

During anesthesia, single-drug sedation often requires a higher dose, leading to hemodynamic suppression and a prolonged time to arousal [1, 2]. Combining anesthetics may reduce medication doses and associated adverse effects, improve cardiovascular stability, and relieve anxiety [3]. Moreover, combining drugs with the same actions enhances the specific activity (additive and synergistic). Synergy refers to the combination of clinical drugs, which is much more effective than the sum of their individual effects [4]. The interaction coefficient represents the degree of synergy [5]. A typical application of synergy in induced anesthesia is the combined use of midazolam with propofol.

In endoscopy, sufentanil reduces discomfort and the cardiovascular response [3]. Remimazolam, a new short-term benzodiazepine anesthetic, metabolizes faster than midazolam with good efficacy and safety profiles [6]. Currently, remimazolam is used for gastroscopy. The drug can also be used with propofol and potent opioids to induce anesthesia in other clinical conditions [7]. For proper use of this drug, the synergy, the synergistic coefficient, and the optimal ratio between remimazolam and propofol must be determined.

Several approaches can be used to study drug interactions. The most classic method is isobologram analysis [4]. The isobologram analysis uses the dose–effect relationship of each drug to derive a set of dose combinations that are expected to give a specified effect level [4]. This study hypothesizes that the interaction between remimazolam and propofol has additive effects. Therefore, the synergistic effects were investigated using isobologram analysis.

### Aim

The study aimed to investigate the synergistic interaction between remimazolam and propofol after sufentanil administration and to determine appropriate dose ratios between remimazolam and propofol.

### Ethics approval

This study was approved by the Clinical Research Ethics Committee of the Second Affiliated Hospital of Hainan Medical University, Haikou, China (approval number LW202101, approval date January 4, 2021). The trial was registered at the Chinese Clinical Trial Registry (ChiCTR2100052425, principal investigator: Song Lyu, registration date: October 26, 2021). Written informed consent was obtained from all patients who participated in the trial.

## Method

### Study design and patients

The study was conducted in the Gastrointestinal Endoscopy Department on January 10, 2021. Inclusion criteria were patients scheduled for gastrointestinal endoscopy who had the American Society of Anesthesiologists (ASA) classification grade I/II [8] and body mass index (BMI) ≤ 30. Exclusion criteria were patients who had (1) anemia (hemoglobin < 90 g/L), (2) albumin level < 30 g/L, (3) platelet count < 50 × 10^9^/L, (4) anticipated difficult airway, (5) significant respiratory or circulatory dysfunction before gastrointestinal endoscopy, (6) severe neuropsychiatric disorders, (7) allergic reactions or contraindications to benzodiazepines, opioids, propofol, flumazenil, naloxone, or drug ingredients, or (8) previously received remimazolam, propofol, or sufentanil.

### Randomization and blinding

The experiment was conducted in two steps. Experiments were performed in groups A and B to determine the median effective dose (ED_50_) of remimazolam and propofol. Experiments were conducted in groups C, D, and E to explore the interaction between the two drugs. Therefore, the group randomization was divided into two steps. The block method was used to randomize groups [9]. The random table was generated from http://www.randomization.com. In the first step, 60 patients were randomly assigned to 14 blocks with a block size of 2 and eight blocks with a block size of 4. The number of randomly generated seeds was 21,404. The second step involved randomly assigning 90 patients to 14 blocks with a block size of 3 and eight blocks with a block size of 6. The number of randomly generated seeds was 27,436. Random codes were concealed in opaque envelopes in the order in which patients were added.

According to groups, the patients were labeled with five different colors by study personnel blinded to grouping. Drug labels were removed after administration. Observers were unaware of groups and drug doses; only the investigator and anesthesiologist knew the details.

### Intervention and measurements

An open intravenous access was established in the patient’s upper extremity in the waiting area. After entering the endoscopy room, the patient was placed in the supine position on the operation bed. Blood pressure, electrocardiogram, and blood oxygen saturation were monitored. The doses of drugs were calculated based on the actual body weight, using specifications based on those of a previous study [10].

Remimazolam (201114AK, Hengrui, Jiangsu, China) was diluted to 0.5 mg/mL, while propofol (2011171, Guorui, Sichuan, China) was diluted to 1, 2, and 10 mg/mL with normal saline (NS). To minimize discomfort, all patients received 0.1 μg/kg of sufentanil (01A10011, Renfu, Sichuan, China) intravenously over 10 s. The cannula was washed with 5 mL of NS. After 90 s, remimazolam and propofol were administered over 10 s according to the group-specific dose. The two drugs were administered at 20-s intervals. After each dose, the cannula was rinsed with 5 mL of NS, and the observation began after 1 min. Hypnosis was achieved when the eyelash reflex was lost [6], and the study was terminated [11]. Because the depth of anesthesia in which the eyelid reflex disappeared was insufficient to prevent the patient’s reaction to the endoscope insertion, additional propofol was administered to deepen the anesthesia before the endoscope insertion.

The ED_50_ of propofol and remimazolam could be affected by sufentanil. The first patient in each group received an ED_50_ dose of remimazolam and propofol based on a previous study [12]. If the eyelash reflex did not disappear, the dose was increased to 1.25 times the given dose. If the reflex disappeared, the dose was reduced to 0.8 times the given dose. After repeating the procedure in several patients, the optimal dose of each drug was determined, which should be around ED_50_ [13, 14].

The patients were divided into group A (remimazolam), group B (propofol), group C (0.25ED_50_remimazolam plus propofol), group D (0.5ED_50_remimazolam plus propofol), and group E (0.75ED_50_remimazolam plus propofol). In the pretest, the remimazolam ED_50_ was estimated to be 0.08 mg/kg. This dose was used as the starting dose in group A to determine the remimazolam ED_50_. The propofol ED_50_ was reported as 0.9 mg/kg when the eyelid reflex was absent [12]. This dose was used as the starting dose in group B to obtain the propofol ED_50_. Using the ED_50_ of propofol obtained from group B as the starting dose, propofol ED_50_ values were recalculated for groups C, D, and E when propofol was administered with remimazolam (the dose of remimazolam was fixed in each group).

### Data collection and outcomes

A data collection form was used to record the patient identification code, sex, age, height, weight, BMI (calculated by height/weight), remimazolam and propofol doses, and the loss of eyelash reflex. Information was entered into the hospital database. The outcomes were ED_50_ of remimazolam and propofol in each group and the interaction coefficients between the two drugs.

### Sample size

The up-and-down approach significantly reduces the subjects required for a study [14]. The gap between the starting dose and the ED_50_ and the individual variability in the dose–response relationship makes it difficult to determine the sample size in advance [13]. The number of subjects must be large enough to meet at least six trials [14]. Increasing the number of trials increases the number of subjects and makes the ED_50_ values closer to the actual values. Therefore, seven trials were chosen to calculate the sample size. Based on the calculation that one test occurs in every four patients, at least 28 subjects are required for seven trials. A total of 30 patients were needed considering a loss of follow-up rate of 0.1.

### Statistical analysis

Age, height, weight, and BMI are represented as median and interquartile ranges. Statistical analyses were performed using SPSS (version 22, IBM, USA). One-way analysis of variance (ANOVA) was used to compare demographic information between groups. *P* < 0.05 was considered statistically significant.

Interval estimates are used for statistical inference, with 95% confidence intervals (95% CI) as criteria for statistical significance. ED_50_ and 95% CI of remimazolam and propofol were calculated using probabilistic regression analysis [13]. The additive line and the 95% CI of the additive line were drawn based on the 95% CI and ED_50_ of remimazolam and propofol [5]. When remimazolam and propofol are administered simultaneously, ED_50_ values can be used to draw a curve. Curves were considered clinically significant if they were below and to the left of the additive line. Interaction coefficients were calculated by algebraic analysis as follows:

$$Interaction\,coefficients = 1\left/\left( {\frac{Dose\,of\,remimazolam}{{remimazolam\,ED_{50} }} + \frac{Dose\,of\,propofol}{{propofol\,ED_{50} }}} \right)\right.$$

## Results

**Fig. 1:**
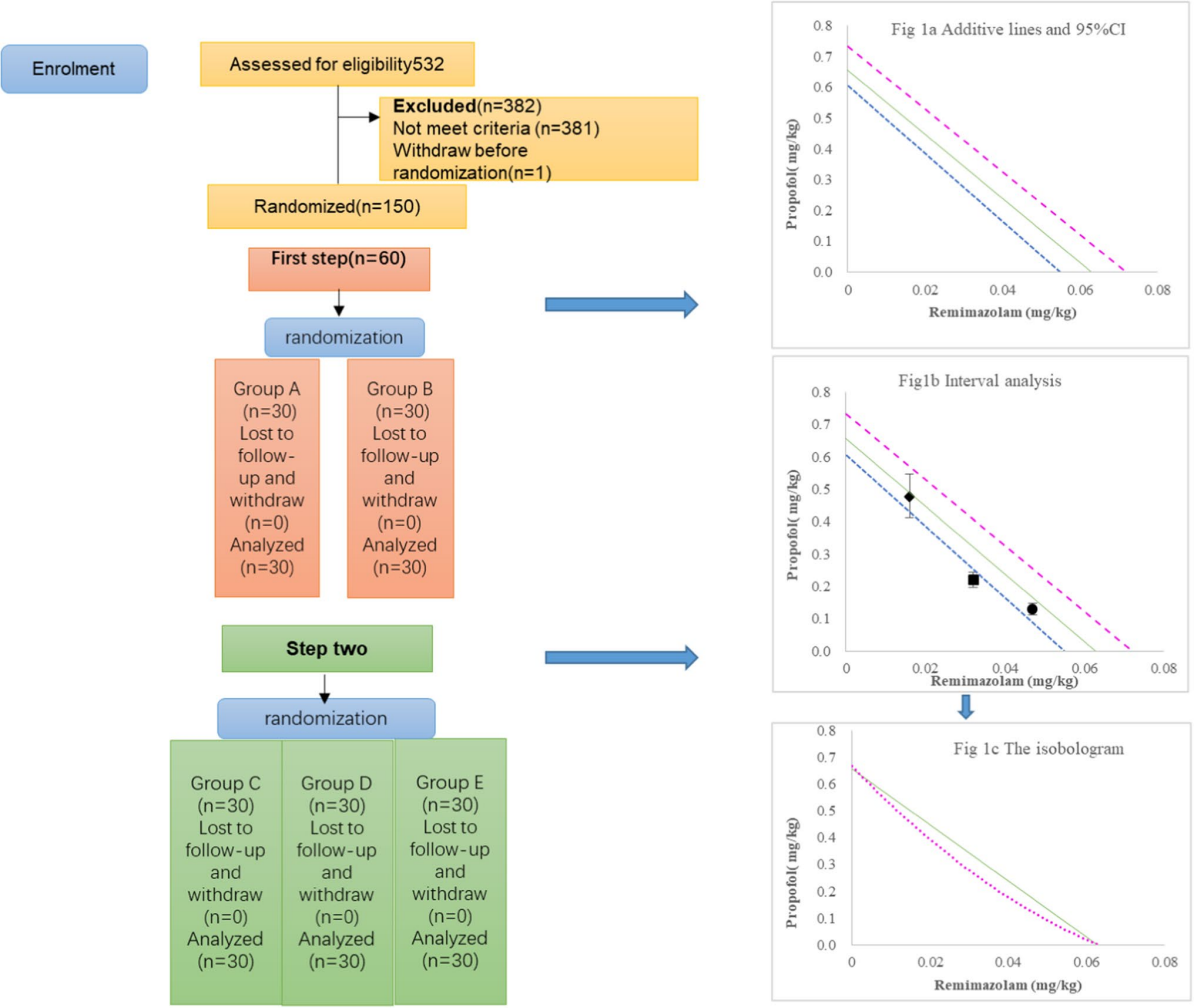
Recruitment, intervention, and analysis of included patients. **a** The additive line and the 95% confidence interval (CI); **b** interval analysis; **c** the isobolographic representation of the combination of propofol and remimazolam after administration of 0.1 µg/kg sufentanil. *Filled diamond* 0.75ED_50_remimazolam + propofol, *filled square* 0.5ED_50_remimazolam + propofol and *filled circle* 0.25ED_50_remimazolam + propofol. The green line represents the line of simple addition. The pink and blue lines are the upper and lower 95% CI of the line of simple addition (**a**). Group D (0.5ED_50_remimazolam + propofol) has a statistically significant synergic effect as the 95% CI of the drug combination is below the blue line (**b**). Isobolographic analysis shows that remimazolam and propofol have a clinically significant synergistic effect as the ED_50_ curve of the drug combination is below the simple additive line (**c**)

A total of 150 patients were enrolled and were randomly divided into groups A to E. (Fig. 1). No significant differences were observed in sex, age, height, weight, and BMI between groups (*P* > 0.05). Details are shown in Table 1.

**Table 1:** Baseline characteristics of the included patients

Characteristics	Group A (n = 30)	Group B (n = 30)	Group C (n = 30)	Group D (n = 30)	Group E (n = 30)
Sex					
Male	11	13	17	19	11
Female	19	17	13	11	19
Age (year), median (IQR)	54 (14)	51 (19)	53 (14)	50 (21)	52 (18)
Weight (kg), median (IQR)	58 (11)	59 (18)	54 (20)	57 (13)	61 (17)
Height (cm), median (IQR)	159 (11)	159 (19)	158 (23)	160 (23)	164 (16)
BMI (kg/m^2^), median (IQR)	23 (3)	24 (3)	23 (2)	22 (2)	23 (4)

*IQR* interquartile range

ANOVA analysis results showed no difference between groups (*P* > 0.05)

Remimazolam ED_50_ in group A was calculated to be 0.063 mg/kg (95% CI 0.055–0.072 mg/kg) when combined with 0.1 μg/kg sufentanil. Propofol ED_50_ in group B was calculated to be 0.672 mg/kg (95% CI 0.606–0.732 mg/kg) when combined with 0.1 μg/kg sufentanil (Fig. 1a). Using the starting dose of 75% of the ED_50_ dose (0.504 mg/kg) obtained in group B, different doses of propofol were administered for group C patients in combination with remimazolam (0.25ED_50_ = 0.016 mg/kg) and 0.1 mg/kg sufentanil to obtain the propofol ED_50_ in group C. Propofol ED_50_ in group C was calculated to be 0.477 mg/kg (95% CI 0.416–0.539 mg/kg). In group D, the starting dose of propofol was 50% of the ED_50_ dose (0.336 mg/kg). Different doses of propofol were administered for group D patients in combination with remimazolam (0.5ED_50_ = 0.032 mg/kg) and 0.1 μg/kg sufentanil. Propofol ED_50_ in group D was calculated to be 0.221 mg/kg (95% CI 0.198–0.244 mg/kg). Interval analysis showed statistically significant synergistic effects between propofol and remimazolam (Fig. 1b).

**Fig. 2:**
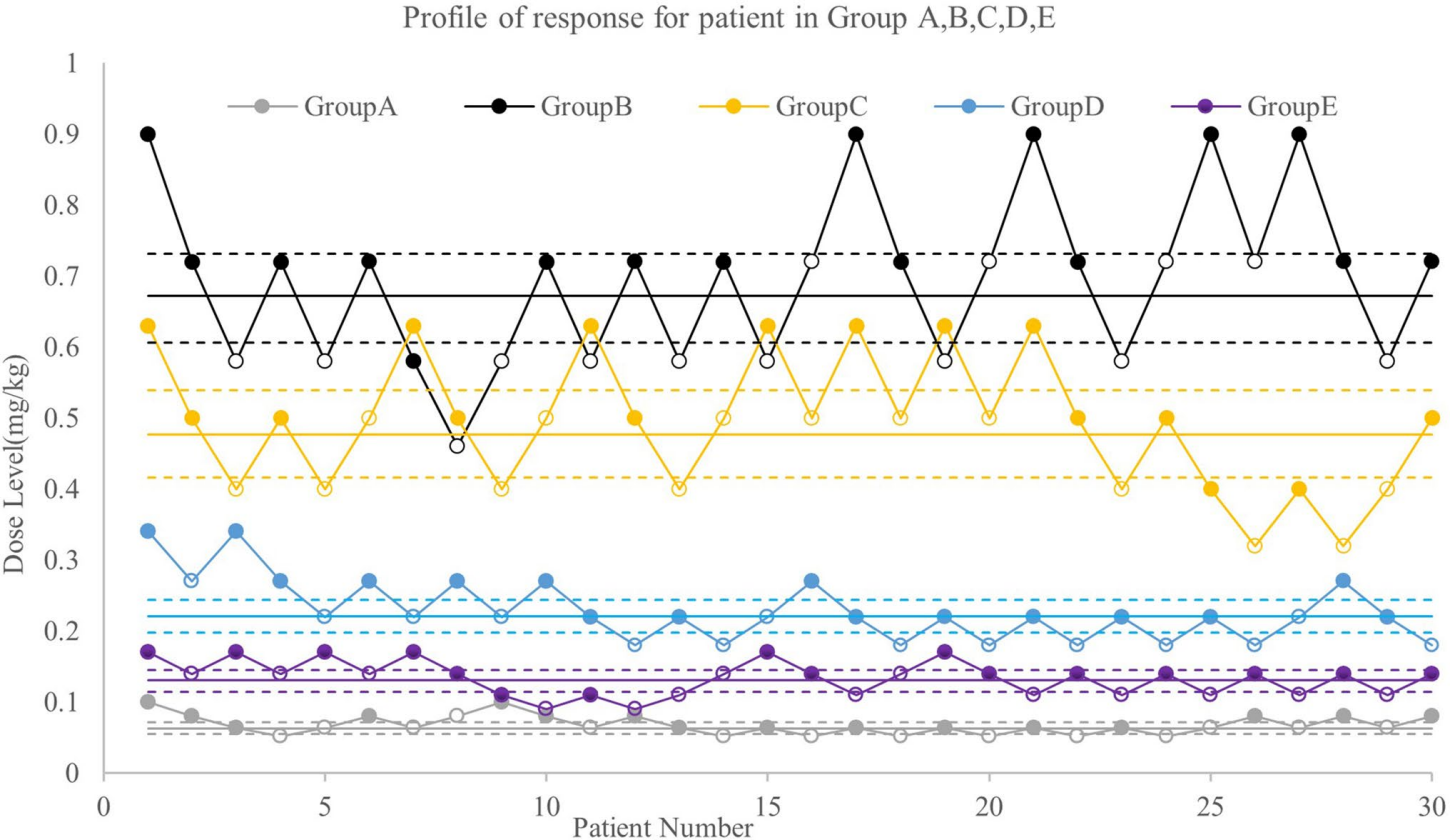
Sequence of dosing in the five groups. Group A: sufentanil + remimazolam, Group B: sufentanil + propofol, Group C: sufentanil + 25%ED_50_remimazolam + propofol, Group D: sufentanil + 50%ED_50_remimazolam + propofol, Group E: sufentanil + 75%ED_50_remimazolam + propofol. The open circles represent the loss of the eyelash reflexs and the solid circles represent the presence of the eyelash reflex. The solid and dotted horizontal lines are the ED_50_ and 95% CI. ED_50_ (median effective dose), 95% CI (95% confidence interval)

Different doses of propofol were administered for group E patients in combination with remimazolam (0.75ED_50_ = 0.047 mg/kg) and 0.1 μg/kg sufentanil. In group E, the starting dose of propofol was 25% of the ED_50_ dose (0.168 mg/kg). Propofol ED_50_ in group E was calculated to be 0.131 mg/kg (95% CI 0.14–0.145 mg/kg). The dose–response curves of the patients in groups A to E are shown in Fig. 2.

The cross-sectional analysis of the isobologram (Fig. 1c) showed a clinically significant synergistic effect between remimazolam and propofol. Algebraic analysis (Table 2) showed that the synergistic coefficients for remimazolam and propofol were 1.04, 1.21, and 1.06, respectively. As shown in Table 2, when the doses of remimazolam and propofol were 0.032 and 0.221 mg/kg (group D), the combination had the highest interaction coefficient of 1.21, with an improved synergistic effect. Thus, the dose ratio of remimazolam to propofol was approximately 1:7.

**Table 2:** Interactions of remimazolam and propofol combined with 0.1 µg/kg sufentanil, evaluated based on eyelash reflex inhibition

Remimazolam		Propofol		Coefficient sum	Expected value of additive action	Interaction coefficient
ED_50_ coefficient	ED_50_ (mg/kg)	ED_50_ coefficient	ED_50_ (mg/kg)			
1	0.063 (0.055–0.072)	0	0	1	1	1
0	0	1	0.672 (0.606–0.732)	1	1	1
0.25	0.016	0.710	0.477 (0.416–0.539)	0.960	1	1.04
0.5	0.032	0.329	0.221 (0.198–0.244)	0.829	1	1.21
0.75	0.047	0.195	0.131 (0.114–0.145)	0.945	1	1.06

Interaction coefficient = 1/coefficient sum

Parentheses show the 95% confidence interval

## Discussion

This study demonstrated a clinical synergistic effect between remimazolam and propofol. When the two drugs are combined, each drug's dose should be reduced. Both remimazolam and propofol have rapid distribution and metabolism. When the dose of one medicine is too small, with quick distribution and metabolism, the process will lead to a rapid decrease in the drug plasma concentration resulting in a rapid reduction in the amount of the drug that can exert a synergistic effect. This is why the ED_50_ of groups C and E are within the 95% CI of the simple additive line. Therefore, to take advantage of the synergistic effect in anesthesia, attention should be paid to the ratio of the two drugs.

According to the results of this study, if the anesthesia strength produced by 1 mg/kg of propofol is to be achieved, only 0.33 mg/kg of propofol and 0.05 mg/kg of remimazolam are needed for the combined administration. During the maintenance period of anesthesia, the two drugs may be continuously infused. It is also recommended to keep the infusion at this ratio.

Drug synergy has been attributed to pharmacodynamic or pharmacokinetic interactions [5]. Unlike midazolam, which is metabolized by cytochrome (CYP) P450 3A4 and is inhibited by many drugs [15], remimazolam is metabolized by tissue carboxylesterase 1 (CES1) enzyme [16]. CES1 is highly expressed in the liver, bile, and lungs. Remimazolam is unlikely to interact for pharmacokinetic reasons. Propofol is metabolized primarily in the liver by CYP2B6 and CYP2C9 [17]. About 70% of propofol is conjugated to glucuronide through uridine 5′-diphosphate (UDP) glucuronosyltransferase, and 29% is hydroxylated to 2, 6-diisopropyl-1,4-quinoline (4-hydroxypropofol). Therefore, the metabolic pathways of propofol and remimazolam are unlikely to be synergistic.

The protein binding rates of remimazolam and propofol are 92% [18] and 98% [19,20], respectively. Propofol causes an increase in midazolam blood concentrations [20]. Whether the synergistic effect between propofol and remimazolam is due to the high protein binding rate still needs further study.

Remimazolam shows slightly higher potency in the α1 subunit of the γ-aminobutyric acid subtype A (GABA-A) receptor. The α1 subunit has been implicated in the sedative, anterograde memory loss, and anticonvulsant effects of benzodiazepines [19]. Propofol acts on the β3-subunit and mediates most of its effects by modulating GABA-A receptors [21]. Therefore, the synergistic effect between remimazolam and propofol may occur at the post-receptor stage, which requires further experimental confirmation.

The results of group A also demonstrated a synergistic effect between remimazolam and opioid analgesics [22–24]. The same data from group B confirmed the synergy between propofol and sufentanil [12]. When remimazolam is combined with sufentanil, the dose of remimazolam should be reduced. In this study, the dose of remimazolam was 0.075 mg/kg when combined with sufentanil to induce sedation.

Remimazolam is less effective at suppressing the pharyngeal reflex [25]. Throat irritation is severe during gastroscope insertion, which requires a combination of opioids and propofol. Studies have shown that midazolam and propofol synergize with alfentanil [9]. The synergistic effect has its clinical value in anesthesia. The synergistic impact confirmed in this study indicates that the combination of sufentanil, remimazolam, and propofol has potential clinical application value.

This study has the following limitations: (1) this is a single-center study with limited representation, (2) there may be stronger interaction ratios around 0.5ED_50_ that require further investigation, (3) the disappearance of the eyelid reflex is only a point of clinical anesthesia, and the interaction among other clinical endpoints requires further investigation, and (4) the occurrence of adverse reactions after the combination of drugs also must be observed.

## Conclusion

Remimazolam and propofol have synergistic clinical effects. A strong synergistic effect was observed when the remimazolam and propofol dose ratio was 1:7 (mg/kg).
